# Large-scale production and antiviral efficacy of multi-target double-stranded RNA for the prevention of white spot syndrome virus (WSSV) in shrimp

**DOI:** 10.1186/s12896-015-0226-9

**Published:** 2015-12-01

**Authors:** Thitiporn Thammasorn, Pakkakul Sangsuriya, Watcharachai Meemetta, Saengchan Senapin, Sarocha Jitrakorn, Triwit Rattanarojpong, Vanvimon Saksmerprome

**Affiliations:** Center of Excellence for Shrimp Molecular Biology and Biotechnology, Faculty of Science, Mahidol University, Bangkok, 10400 Thailand; Department of Biochemistry, Center of Excellence for Molecular Biology and Genomics of Shrimp, Faculty of Science, Chulalongkorn University, Bangkok, Thailand; National Center of Genetic Engineering and Biotechnology, (BIOTEC), Thailand Science Park, Pathum Thani, 12120 Thailand; Department of Microbiology, Faculty of Science, King Mongkut’s University of Technology Thonburi, Bangkok, 10140 Thailand

**Keywords:** Co-cultivation, White spot syndrome virus, dsRNA, Shrimp, VP28, WSSV051

## Abstract

**Background:**

RNA interference (RNAi) is a specific and effective approach for inhibiting viral replication by introducing double-stranded (ds)RNA targeting the viral gene. In this study, we employed a combinatorial approach to interfere multiple gene functions of white spot syndrome virus (WSSV), the most lethal shrimp virus, using a single-batch of dsRNA, so-called “multi-WSSV dsRNA.” A co-cultivation of RNase-deficient *E. coli* was developed to produce dsRNA targeting a major structural protein (VP28) and a hub protein (WSSV051) with high number of interacting protein partners.

**Results:**

For a co-cultivation of transformed *E. coli*, use of Terrific broth (TB) medium was shown to improve the growth of the *E. coli* and multi-WSSV dsRNA yields as compared to the use of Luria Bertani (LB) broth. Co-culture expression was conducted under glycerol feeding fed-batch fermentation. Estimated yield of multi-WSSV dsRNA (μg/mL culture) from the fed-batch process was 30 times higher than that obtained under a lab-scale culture with LB broth. Oral delivery of the resulting multi-WSSV dsRNA reduced % cumulative mortality and delayed average time to death compared to the non-treated group after WSSV challenge.

**Conclusion:**

The present study suggests a co-cultivation technique for production of antiviral dsRNA with multiple viral targets. The optimal multi-WSSV dsRNA production was achieved by the use of glycerol feeding fed-batch cultivation with controlled pH and dissolved oxygen. The cultivation technique developed herein should be feasible for industrial-scale RNAi applications in shrimp aquaculture. Interference of multiple viral protein functions by a single-batch dsRNA should also be an ideal approach for RNAi-mediated fighting against viruses, especially the large and complicated WSSV.

## Background

White spot syndrome virus (WSSV), a major pathogen with high infectivity and mortality, has been a serious threat for penaeid shrimp aquaculture in the past two decades. WSSV is a large double-stranded DNA virus with the approximate genome size of 300 kbp [1–3]. Most of their putative translated gene products have no homology to other proteins from viruses or host cells. The uniqueness of WSSV therefore classified the virus into its own family *Nimaviridae* and genus *Whispovirus* [4]. Several aspects including morphology and pathogenicity of WSSV have been intensively studied to seek prevention and therapeutic treatment. The viral control strategies were included administration of recombinant WSSV proteins and DNA vaccine based constructs [1, 5–8]. Application of immunostimulants were also introduced to shrimp to fight against WSSV infection [9, 10]. Nevertheless, no practical and effective methods have been established to control WSSV yet.

Application of RNA interference (RNAi) or double-stranded (ds)RNA-mediated viral inhibition has been shown to be a promising anti-WSSV strategy [11–15]. In this study, we proposed a combinatorial approach to interfere multiple WSSV gene expression using a single batch of dsRNA (hereafter called “multi-WSSV dsRNA”). Targeting multiple viral targets by dsRNA could possibly result in additive inhibition; however, more importantly, this approach should lower the chance of viral escape that needs to have multiple resistance mutations within the dsRNA targets occurred simultaneously [16]. The target viral genes in this study include a major structural protein (VP28) and a hub protein (WSSV051). VP28 is involved in the viral entry to shrimp cells, and injection of dsRNA corresponding to VP28 was shown to effectively protect shrimp against the virus [11, 13, 14]. Oral administration of VP28-specific dsRNA was demonstrated as a potential therapeutic method by improving shrimp survival rate after WSSV challenge [17]. WSSV051, also known as structural protein VP55, has been recently identified as one of the hub proteins from the WSSV protein-protein interaction network [15]. The hub function is to hold the proteins together in the network therefore knock-down of WSSV hubs would be expected to collapse WSSV functions, and silencing this gene by specific dsRNA could delay shrimp mortality after WSSV infection [15].

Here, a co-cultivation of RNase-deficient *E. coli* was developed to produce multi-WSSV dsRNA, and large-scale production of the multi-WSSV dsRNA was optimized through a glycerol feeding fed-batch fermentation. Feed pellets formulated with the multi-WSSV dsRNA were prepared according to the method described by Saksmerprome et al. [18], and their antiviral efficacy was also examined.

## Methods

### Co-cultivation of two strains of RNase-deficient *E. coli* to produce dsRNA targeting multiple WSSV genes

Construction of hairpin expression vector targeting VP28 (GenBank no. AY422228.1, nucleotides 8–189) was developed according to the method described by Saksmerprome et al. [19]. The plasmid encoding WSSV-VP28 of 181-bp was used as a template for PCR. Primers used for amplification of DNA template for dsRNA-VP28 synthesis are VP28F (5′ TTT CTT TCA CTC TTT CGG TCG T 3′) and VP28R1 (5′ GCC TGA TCC AAC CTC AGC AGT C 3′). The conditions for PCR amplification were as follows: 3 min at 94 °C, 35 cycles of 30 s at 94 °C, 30 s at 53 °C and 30 s at 72 °C and extension at 72 °C for 5 min. The other construct targeting WSSV051 (GenBank no. AF440570, nucleotides 28034–28316) was performed with modified protocols from Sangsuriya et al*.* [15]. An amplified amplicon of 393 bp was obtained from a PCR reaction using specific primers: 051siF (5′ TTC AGG GCG GCT ATC TTA TG) and 051siR2 (5′ TCA TCT TCT TCC ATG ACA TC3′) and DNA extracted from WSSV-infected gill tissues as template. The conditions for PCR amplification were as follows: 3 min at 94 °C, 35 cycles of 30 s at 94 °C, 30 s at 55 °C and 30 s at 72 °C and extension at 72 °C for 5 min. The PCR product was then purified and cloned in a sense orientation downstream of the T7 promoter of pDrive vector (QIAGEN). Subsequently, an amplicon of 283 bp obtained using a primer set harboring XbaI and HindIII restriction sites (XbaI-051siF: 5′ GC TCTAGA TTC AGG GCG GCT ATC TTA 3′ and HindIII-051siR3: 5′ AC AAGCTT AAA GAA AAC CCC TTC TGG 3′) was cloned in an antisense orientation downstream of the first fragment. The hairpin constructs containing both sense and antisense strands were then verify by DNA sequencing.

Each recombinant plasmid was transformed into RNase III-deficient *E. coli* HT115 (DE3) for long dsRNA production. Two types of cultivation medium, Luria Bertani (LB) and Terrific broth (TB), were used to achieve the optimal production of bacteria cells and dsRNA. LB medium consisted 1 % (w/v) tryptone, 0.5 % (w/v) yeast extract, and 1 % (w/v) NaCl. Composition of TB medium was 1.2 % (w/v) tryptone, 2.4 % (w/v) yeast extract, 0.72 M potassium phosphate (dibasic), 0.17 M potassium phosphate (monobasic), and 0.4 % (v/v) glycerol. For a starter culture, a single transformant colony for each dsRNA population was inoculated into 3 mL of either LB or TB medium in the presence of 100 μg/mL ampicillin and 12 μg/mL tetracycline. The culture was shaken at 250 rpm, 37 °C until OD_600 nm_ of 3.5–4.0 was obtained (approximately 16 h). Co-inoculation was subsequently performed at the ratio 1:100 (v/v) in fresh medium. The co-culture was shaken for 8 h at 37 °C, and was collected for analysis.

### Fermentation processes for large-scale production of multi-WSSV dsRNA

Large-scale production of multi-WSSV dsRNA was conducted in a 10-L bioreactor at Biochemical Engineering and Pilot Plant Research and Development Unit (BEC) of KMUTT, Thailand. The starter culture was prepared as described above. Co-inoculation was performed at the ratio 1:40 (v/v) in 5-L fresh medium containing 100 μg/mL ampicillin and 12 μg/mL tetracycline. For both batch and fed-batch fermentation, temperature was maintained at 37 °C and pH was kept at 7.0 using 1 M NH_4_OH. Dissolved oxygen (DO) was controlled at 30 % of air saturation. Batch fermentation was run for 8 h, and 50 ml of the culture were collected for analysis. The remaining culture underwent the fed-batch process by feeding 500 g/L of glycerol as the carbon source at the rate of 2.9 mL/h. Fed-batch fermentation was continued until sequential increasing of DO (%) value was indicated as the decline phase (or cultivation time of 30 h), and 50 mL of fed-batch culture were collected for analysis.

Cell concentrations (CFU/mL) obtained from lab-scale and large-scale fermentation were determined by measuring UV absorbance at 600 nm, and 1 OD_600nm_ is equivalent to 10^9^ CFU/mL. RNA extraction was performed using phenol-chloroform method [20]. Reverse transcription (RT)-PCR and 1.5 % agarose gel electrophoresis were performed to indicate the presence of WSSV-specific dsRNA. The primer pairs for detection of WSSV05-dsRNA are XbaI-051siF and HindIII-051siR3, for detection of VP28-dsRNA are VP28F and VP28R1. The RT-PCR conditions to detect WSSV051-dsRNA were as follows: 5 min at 50 °C, 5 min at 94 °C, 35 cycles of 10 s at 94 °C, 30 s at 55 °C and 30 s at 68 °C and extension at 68 °C for 5 min, whereas VP28-dsRNA detection were as follows: 5 min at 50 °C, 5 min at 94 °C, 35 cycles of 10 s at 94 °C, 30 s at 53 °C and 30 s at 68 °C and extension at 68 °C for 5 min. Nuclease digestion experiments using RNase A and RNase III were performed as described in Saksmerprome et al*.* [19] to examine quality and integrity of each dsRNA production. The amount of dsRNA was quantitated by measuring UV absorbance at 260 nm, and dsRNA concentration extracted from 1 mL culture was calculated in μg/μl.

### Feed preparation

Four formulas of feed, with different types and doses of dsRNA, were tested; 1) 6 mg of WSSV051-dsRNA, 2) 6 mg of VP28-dsRNA, 3) 6 mg of multi-WSSV dsRNA and 4) 12 mg of multi-WSSV dsRNA. For detailed feed preparation, the commercial shrimp feed (OMEG 1704 S, BETAGRO) was ground using a blender. One kilogram of mashed feed was mixed with 500-mL of the co-culture thoroughly. Feed mixture was pelleted by pressing through 10-mL syringe (2 mm in diameter), and the feed pellets were dried at 60 °C in hot air oven for 24 h. To indicate the presence dsRNA in feed, extracted RNA from 0.1 g feed pellets were subjected to RT-PCR using WSSV051- and VP28-specific primers. The same procedure was repeated on the formulated feed kept at 20–25 °C for 7 months to examine dsRNA stability in feed.

### Oral administration of multi-target dsRNA and WSSV challenge

*Penaeus vannamei* shrimp with the average size of 3–5 g were acclimatized for 3 days in 15 ppt of artificial seawater with aeration, and were arbitrarily divided into 6 groups (*n* = 10) with 3 replicates of each group. Four groups received the dsRNA-formulated feed, while the WSSV-positive and negative controls received commercial feed without modification. Feeding dose rate was at 4 % of their body weight, and shrimp were fed twice a day. Individual 5-g shrimp in groups that received single- and double-dose formulas would receive 1.2 and 2.4 μg dsRNA per meal at the most, respectively. After 5-day feeding, all animals, except the WSSV-negative control group, were injected with 10^5^ copies of WSSV (LD_50_ = 4 days) per shrimp. The animals were fed continuously with the assigned feed for the next 7 days. Shrimp mortality of each group was recorded, and the average time to death was calculated accordingly. Data on average time to death was analyzed by t-test analysis using SPSS18.0 software, *P < 0.05*.

## Results and Discussion

### A single co-cultivation with Terrific Broth medium resulted in high cell density and dsRNA yield

The effect of culture medium on productivity of *E. coli* expression system has been studied by varying various media to achieve the optimal production of bioactive compounds [21, 22], indicating the need for optimization of culture conditions when attempting cultivation of new species. Previous work by our group demonstrated the production of dsRNA against shrimp viruses in RNase-deficient *E. coli* using LB broth medium [18, 19, 23, 24]. A single population of dsRNA against an individual shrimp viral gene is produced in the range of a few micrograms per 100 mL *E. coli* culture under a lab-scale experiment. In this study, we tested to see if use of Terrific broth (TB), a culture medium that is commonly used in culture conditions for protein overexpression [22, 25, 26] would enhance the productivity of co-cultivated bacteria as compared to the original condition with LB broth. For a small-scale cultivation, final cell density (CFU/mL) and dsRNA production (μg/mL culture) in the culture with TB medium were both approximately 2x those of the culture with LB broth (Table 1). The enhanced cell growth and dsRNA yield under TB condition could be explained as follows. TB is a nutritionally rich medium containing a 4.8-time higher yeast extract relative to LB broth. Yeast extract is a main component for the growth of microorganism. It contains nitrogenous compounds, carbon, sulfur, trace nutrients, vitamin B complex and other important growth factors. The additional 0.4 % glycerol is also provided in TB broth as an extra carbon source. Moreover, TB medium contains K_2_HPO4 and KH_2_PO4 which function as buffer medium, therefore the pH of the TB medium is maintained to optimize culture condition during the cell growth [21, 22, 25, 27].

**Table 1:** Determination of cell concentrations and dsRNA yields obtained from a single co-cultivation of RNase-deficient *E. coli*

Production scale	Type of process	Medium	Cell density (×10^9^ CFU/mL^a^)	dsRNAyield (μg/mL^a^)	Incubation time (hr.)
Laboratory	-	LB	2.4 ± 0.2	2.6 ± 0.8	8
	-	TB	4.5 ± 0.1	6.2 ± 0.2	8
Large	Batch	TB	21.1 ± 2.9	3.4 ± 0.5	8
	Fed-batch	TB	36.2 ± 4.5	95.0 ± 21.5	30

^a^1 mL of bacteria culture

### Fed-batch glycerol feeding strategy for large-scale production of multi-WSSV dsRNA

**Fig. 1:**
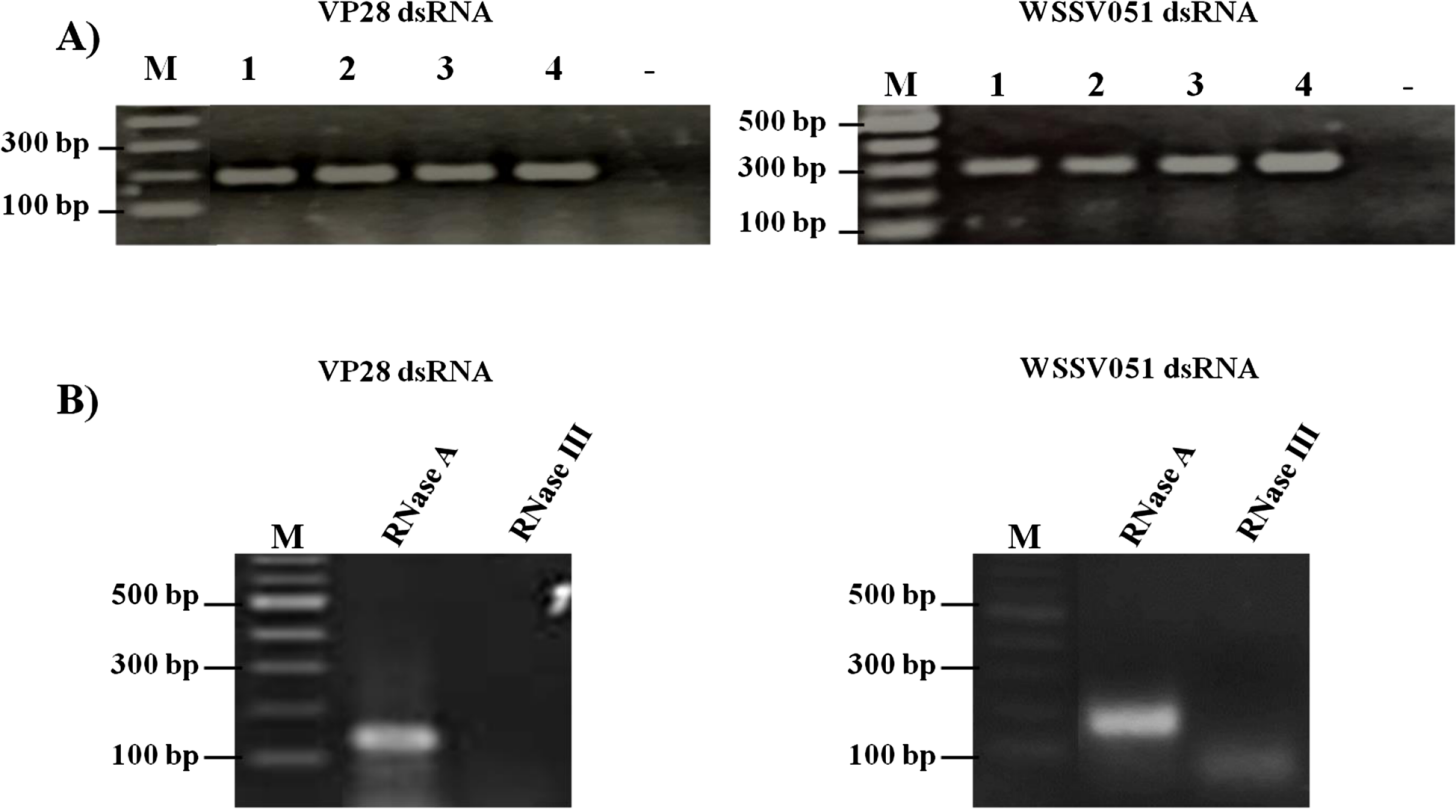
**a** Identification of multi-WSSV dsRNA in a single-batch culture by RT-PCR. Lanes M, 2log marker; lanes 1–2, laboratory-scale production with LB and TB medium, respectively; lanes 3–4, large-scale production under batch and fed-batch processes, respectively; Lane -, negative control using DEP-C water as template. **b** Integrity analysis of individual single-targeted dsRNA from bacterial cells. *E. coli* HT115 expressed WSSV051 and VP28 dsRNA were subjected for dsRNA extraction. The respective dsRNAs were characterized by nuclease treatments using RNase A and RNase III for digestion of the ssRNA and dsRNA, respectively

Two fermentation processes in 10-L bioreactor were employed using TB medium for large-scale production of multi-WSSV dsRNA. For a conventional batch fermentation, the final cell density (CFU/mL) at 8-h cultivation time significantly increased, although dsRNA yield (μg/mL) was half of that obtained in the lab-scale cultivation with TB broth (Table 1). Without additional supplements under the continuous fermentation process, lack of nutrients in high-density culture could limit dsRNA production. In addition, formation of inhibitory factors, such as acetate by-product of aerobic fermentation in the presence of high concentration of carbon source, could have negative effects on growth and *E. coli* and productivity of its products [28–30]. Slow glycerol feeding fed-batch fermentation with controlled dissolved oxygen and pH stability is recommended to improve high cell density and product yields by maintaining the optimal specific growth rate during process [28, 29]. The propagation technique is widely used for production of various bioactive products, including DNA plasmids for vaccine and recombinant proteins [25, 31, 32]. As shown in Table 1, cell density of 36.2× 10^9^ ± 4.5× 10^9^ CFU/mL was obtained at 30-h fed-batch process, producing approximately 95.0 ± 21.5 μg of multi-WSSV dsRNA. The amount of dsRNA as determined from the 1-mL fed-batch culture was almost 30 times higher than the yield obtained under the batch fermentation. RT-PCR followed by 1.5 % agarose gel electrophoresis indicated the presence of dsRNA targeting VP28 (181 bp) and dsRNA targeting WSSV051 (283 bp) in all experiments (Fig. 1a). Evidence for double-stranded nature of the synthesized dsRNAs was obtained from nuclease digestion experiments using RNase A and RNase III depicted in Fig. 1a. Both VP28- and WSSV051-dsRNA were proved to be genuine since they were digested by RNase III but not by RNase A (Fig. 1b).

### Oral administration of feed formulated with multi-WSSV dsRNA reduced shrimp mortality and delayed time to death after WSSV challenge

**Fig. 2:**
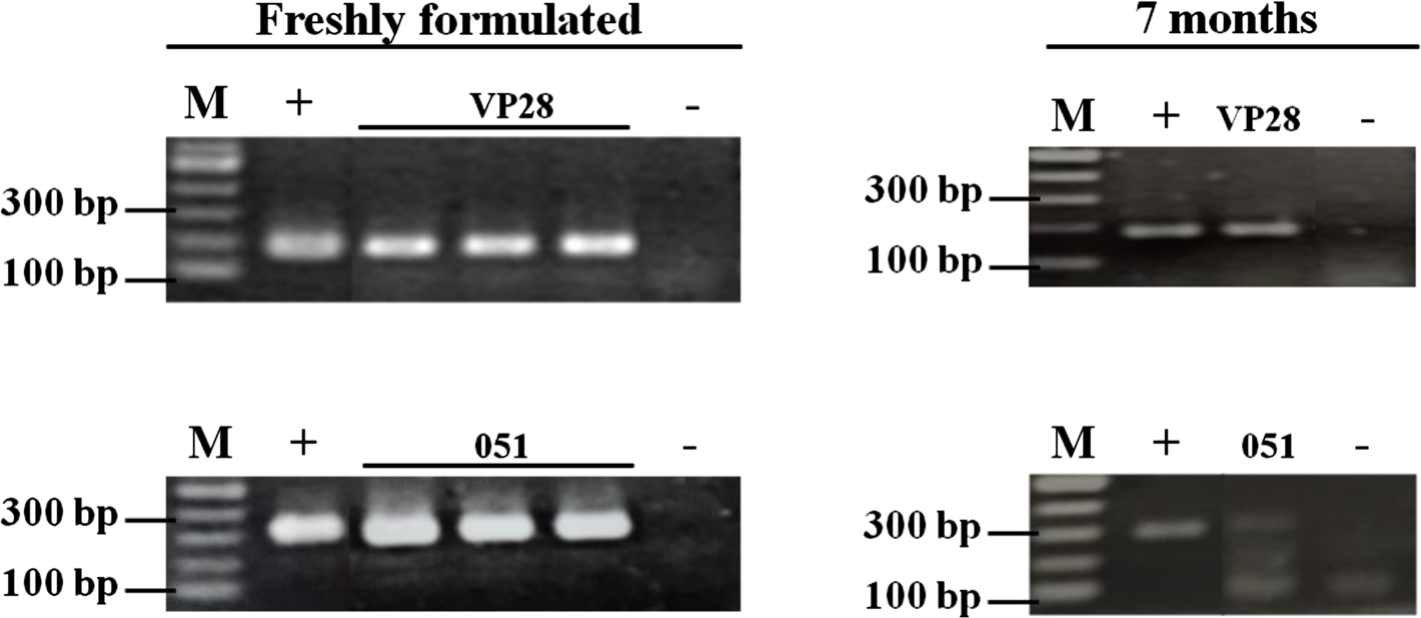
Detection of multi-WSSV dsRNA in the freshly-formulated feed and the formulated-feed stored at 20–25 °C for 7 months. Lanes VP28, VP28-dsRNA; 051, WSSV051-dsRNA; +, positive control using plasmid expressing hairpin VP28 and WSSV051 as templates; −, negative control using DEP-C water as template; M, 2-log DNA ladder

**Fig. 3:**
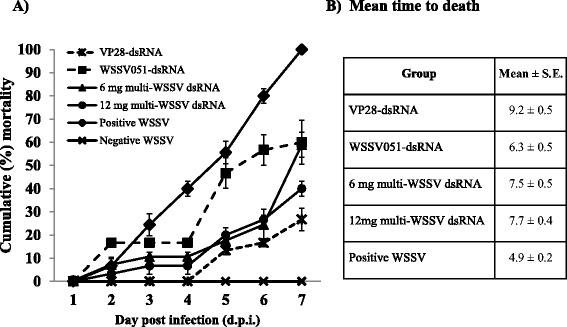
**a** Cumulative mortality rate of shrimp in each group after WSSV challenge. **b** Calculated mean time to death of each group

To test the protective efficacy against the virus of multi-WSSV dsRNA relative to a single gene-targeted dsRNA, feed were formulated to contain single (VP28 or WSSV051) and multiple types (multi-WSSV) with the same quantity of total dsRNA (6 mg each per 1-kg feed). The multi-WSSV formulated feed kept at room temperature for 7 months was examined by RT-PCR, and was found to still maintain both VP28- and WSSV051-dsRNA (Fig. 2). All types of the formulated feed were given to shrimp for 5 days prior to WSSV challenge. The dsRNA-treated groups were monitored in parallel to the control animals for their mortality rate (Fig. 3a) and average time to death, i.e. time when the highest accumulated mortality observed (Fig. 3b). As expected, the WSSV-positive control group showed mortality at 1 day post infection (dpi), and cumulative mortality reached 100 % by the 7th dpi, whereas no cumulative morality observed in the negative-WSSV group at the end of experiment. Comparative efficacy of VP28- and WSSV051- and multi-WSSV dsRNA was also investigated at the same dsRNA quantity per kg feed. WSSV051-dsRNA group showed cumulative mortality of 60 % at 7 dpi, and average time to death of 6 d., affording the least protection among the three groups examined in this study. Viral protective effect of multi-WSSV dsRNA appeared to be intermediate between those of VP28- and WSSV051-dsRNA. The variation of antiviral efficacy might be due to the different targeted genes and their functions in host cells. VP28 is functionally involved in entry step into shrimp cells therefore suppression of this gene resulted in inhibition of viral infection. Although WSSV051 was identified as hub protein, its actual function in shrimp is not yet revealed. To increase efficiency of viral inhibition by WSSV051 dsRNA alone, its higher quantity might be required.

Several WSSV genotypes have been extensively revealed, and this genetic variation is essential to describe viral epidemiology and evolutionary (see review by Shekar M et al. [33]). For instance, the complete genomes of three strains of WSSV-TH (AF369029), WSSV-CN (AF332093) and WSSV-TW (AF440570) were reported, indicating the genetic variations including (i) a large deletion of 13.2 kb in the WSSV-TH; (ii) a genetically variable region found in the WSSV-TH; (iii) an insert of transposable elements in the WSSV-TW; (iv) variation in the number of repeat units within homologous regions and direct repeats; (v) insertions or deletions of single nucleotide mutations and single nucleotide polymorphisms [34]. The differences in genetic content have also been accounted for viral virulence [35–37]. Moreover, mixed-genotypes of WSSV have been investigated in both experimental and natural infections [35, 36, 38]. Therefore, to combat WSSV infection that might cause by different strains and virulence, multiple targets would be a better approach to control viral escape.

Dose dependent effect of dsRNA was also assessed by doubling dose of multi-WSSV in the last formula (12 mg per 1-kg feed). Throughout the experimental time, % mortality of the groups received multi-WSSV dsRNA, at either single or double dose, was significantly lower than that of the WSSV-positive group that did not receive any dsRNA. Average time to death of both single- and double-dose multi-WSSV dsRNA groups were delayed to 7–8 days, while that of the viral positive control was approximately 5 days. Increasing dose of multi-WSSV, from 6 to 12 mg/kg feed, slightly reduced % mortality. It would be interesting to see if significant dose-dependent effect of multi-WSSV dsRNA would be observed under a farm-scale experiment as previously reported by Saksmeprome et al*.* [18].

## Conclusions

This report described the methodology on using co-cultivation approach for large-scale production of dsRNA targeting multiple WSSV genes. First, a single co-cultivation with Terrific Broth medium, where RNase-deficient bacteria expressing different types of dsRNA are simultaneously inoculated, is efficient and time-saving for production of multi-target dsRNA. For large-scale production, fed-batch fermentation with glycerol feeding should be suitable for industrial use of RNAi in shrimp aquaculture. The amount of dsRNA as determined from fed-batch culture was almost 30 times higher than the yield obtained under the conventional batch fermentation. Interim analysis showed that oral application of multi-WSSV dsRNA significantly reduced % shrimp mortality and delayed time to death relative to the WSSV positive control. Despite the intermediate effect of the multi-WSSV dsRNA, compared to the single-targeted dsRNA (VP28 and WSSV051), the use of multiple-target dsRNA should still be encouraged for better controlling the complex WSSV with large genetic variations. Sequence analysis of individual dsRNA components may be necessary to minimize intermolecular interference among them when designing multiple-targeted dsRNA for optimal silencing effect.

## Abbreviations

%: Per cent
bp: Base pair
CFU: Colony forming unit
°C: Degree centigrade
DEP-C water: Diethylpyrocarbonate water
dsRNA: Double stranded RNA
mL: Milliliter
M: Molar
μl: Micro liter
ng: Nano gram
nm: Nano metre
OD: Optical density
RT-PCR: Reverse transcriptase-polymerase chain reaction
rpm: Revolution per minute
v/v: Volume by volume
